# Estrogen Receptor 2b Is Involved in Regulating Gonadotropin-Inhibitory Hormone Expression During Early Development in Zebrafish

**DOI:** 10.3390/ani16030444

**Published:** 2026-02-01

**Authors:** Wei Peng, Bolan Zhou, Yunsheng Zhang, Lili Hu, Liangguo Liu

**Affiliations:** 1 College of Life and Environmental Sciences, Hunan University of Arts and Science, Changde 415000, China; yskaoyan@163.com (Y.Z.); hulili@huas.edu.cn (L.H.); llg1818@126.com (L.L.); 2 Hunan Provincial Key Laboratory for Molecular Immunity Technology of Aquatic Animal Diseases, Changde 415000, China

**Keywords:** *GnIH*, estrogen receptors, gene knockout

## Abstract

Gonadotropin-inhibitory hormone (*GnIH*) is a neuropeptide involved in the regulation of reproductive function in vertebrates. It is able to inhibit the synthesis and secretion of *GnRH* and Gth in the HPG axis. However, the regulatory role and mechanism by which current gonadal steroid hormones regulate *GnIH* are still unclear. In this study, transcription factor binding site analysis was performed on the promoter sequence of zebrafish *GnIH*. Whereafter, transgenic zebrafish (*GnIH*: mCherry) accurately labeled *GnIH* and *esr2b* knockout zebrafish, which were constructed previously, were used to explore the regulation between estrogen and *GnIH*. In vitro exposure of wild-type zebrafish embryos and transgenic zebrafish embryos to estradiol showed that 10 μM estradiol significantly increased the transcription level of *GnIH*. However, both 10 μM and 50 μM estradiol significantly increased the transcription level of *GnIH* in *esr2b* knockout zebrafish. We compared the expression levels of *GnIH* in *esr2b* knockout zebrafish and wild-type zebrafish at different developmental stages (48 hpf–120 hpf). The results showed that from 96 hpf, the expression level of *GnIH* in *esr2b* knockout zebrafish was significantly higher than that in wild-type zebrafish, indicating that *esr2b* is involved in the negative regulation of *GnIH*, and this regulatory relationship is established on the fourth day of zebrafish development.

## Simple Summary

In this study, Tg (*GnIH*: mCherry) and *esr2b* knockout zebrafish were constructed to explore the regulation between estrogen receptors and gonadotropin-inhibitory hormone (*GnIH*). Our work showed that *GnIH* neuropeptide expression location overlaps with that of *Hcrt* at 36 hpf and 72 hpf. Estrogen treatment experiments showed that an appropriate dose of E2 is able to induce *GnIH* mRNA levels. High-dose E2 will induce a feedback mechanism. *Esr2b* knockout led to increased *GnIH* mRNA levels in zebrafish embryos and it started on the fourth day. In a word, estrogen receptors are able to regulate *GnIH* expression, but *esr2b* is involved in negative regulation.

## 1. Introduction

Gonadotropin-inhibitory hormone (*GnIH*) is a short peptide composed of twelve amino acids that was isolated from the hypothalamus of Japanese quail by the Japanese scholar Tsutsui [1]. Subsequently, homologous genes for *GnIH* have been identified in different fish, including goldfish, Nile tilapia, European eel, Atlantic salmon, Medaka, Zebrafish, and common carp [2,3,4,5,6,7]. *GnIH* is able to negatively influence the reproduction of vertebrates through the hypothalamic–pituitary–gonadal (HPG) axis. Micro-osmotic pumps containing *GnIH* peptides were implanted under the skin of male Japanese quail, and after two weeks of treatment, the mRNA expression of the alpha and *LH* beta subunits of gonadotropins in the pituitary gland of the quail was significantly decreased [8]. The mRNA expression of the *FSH* beta subunit also decreased, and the levels of LH and testosterone in the blood were significantly reduced [8]. Results in hamsters showed that the secretion of LH was significantly inhibited after intracerebroventricular or subcutaneous injection of *GnIH* peptides [9]. The inhibitory effect of *GnIH* on gonadotropins may be achieved by directly affecting the secretion activity of *GnRH*. In the brain of European starlings, Ubuka and colleagues found that *GnIH* and *GnRH* nerve fibers were directly connected [10]. In addition, *GnIH* receptors were found to be expressed in GnRH-secreting cells [10]. Similarly, the direct connection between *GnIH* nerve fibers and *GnRH* was detected in the septal regions, preoptic area, and anterior hypothalamic area of rodents’ (mice, rats, and hamsters) brains [8]. Electrophysiological experiments also showed that the injection of *GnIH* peptides into the mouse brain could directly inhibit the activity of 41% of *GnRH* neurons [11]. In summary, current research indicates that *GnIH* may negatively regulate vertebrate reproductive activity by inhibiting the secretion activity of *GnRH* neurons or directly inhibiting the secretion of gonadotropins in the pituitary gland.

Estrogen is a type of steroid hormone produced by the ovaries through the catalysis of aromatase P450 from testosterone [12]. Estrogen plays an important role in ovarian development and maintenance of secondary sexual characteristics in female vertebrates. The synthesis and secretion of key factors *GnRH*, *FSH*, and *LH* in the hypothalamic–pituitary–gonadal axis are regulated by estrogen feedback [13]. The main function of estrogen is to bind and activate its receptor in order to regulate downstream gene expression. In rodents, two types of estrogen receptors were identified: estrogen receptor α(ERα) and estrogen receptor β [14,15]. In zebrafish, four kinds of estrogen were identified: *esr1* (ERα), *esr2a* (ER βII), *esr2b* (ER βI), and *gper* [16]. To analyze the role of different estrogen receptors in teleosts, gene editing technology TALENS or CRISPR-Cas9 were used in ER knockout zebrafish construction. In *gper* knockout female zebrafish, plasma levels of vitellogenin were reduced significantly and follicle development was retarded [17]. *Esr1*, *esr2a*, and *esr2b* single knockout resulted in a male-biased sex ratio (70–85%), but reproductive development and function were normal [18]. It was also reported that *esr2b* knockout leads to embryo development delay and a mortality rate increase during zebrafish development [19]. qPCR and in situ hybridization results demonstrated a significant downregulation of *cyp19ab1b* expression in *esr2b* knockout embryos compared to wild-type embryos [19]. Meanwhile, few study have focused on the function of estrogen receptors during early embryo development. The MO technique is a widely used approach for investigating gene function during the early stages of embryonic development in zebrafish. Griffin designed all *esr1*, *esr2a*, and *esr2b* MOs. Single or combined MOs were injected into zebrafish embryos [20]. Both *esr1* and *esr2b* MOs blocked estradiol induction of vitellogenin and ERα mRNAs. Only *esr2b* MO was able to block the induction of cyp19a1b mRNA through estrogen treatment [20]. It was also reported that *esr2a* is involved in neuromast development in zebrafish [21]. *Esr2a* MO activated the Notch signaling pathway, leading to abnormal hair cell development [21].

In ovariectomized mouse, subcutaneous administration of 17β-estradiol for 4 days by implanting silastic capsules significantly reduced *GnIH* mRNA levels [22]. Evidence from dual-label immunohistochemistry showed approximately 20% of the *GnIH* neurons expressed estrogen receptor (ER)-α, but not *ERβ* [8]. In female hamsters, ER-α mRNA was also found in *GnIH* immunoreactive cells [8]. Estrogen treatment in ovariectomized hamster increased c-fos labeling in *GnIH* neurons [8].

Although estrogen was found to influence *GnIH* expression in rodent brains, reports are rare, and the mechanisms are still unclear. In this study, Tg (*GnIH*: mCherry) transgenic zebrafish and *esr2b* knockout zebrafish were established to assess the relationship between estrogen and *GnIH* expression.

## 2. Materials and Methods

### 2.1. Zebrafish

Different zebrafish lines (wild-type\transgenic\gene knockout zebrafish) were raised at a constant temperature of 28 degrees Celsius and under a 12 h:12 h light–dark cycle. Zebrafish embryos were obtained through natural breeding of female and male zebrafish. To facilitate subsequent in situ hybridization experiments and fluorescence observation, 0.003% (*w*/*v*) of 1-phenyl-2-thiourea was added to the water when the zebrafish embryos developed to 24 h to inhibit pigment formation. All procedures were conducted in accordance with the Guiding Principles for the Care and Use of Laboratory Animals and were approved by the Special Committee of Science Ethics, Academic Committee of Hunan University of Arts and Sciences (2022090733).

### 2.2. Drug Treatment

17β-estradiol(E2) (Sigma, St. Louis, MO, USA) used for drug treatment was purchased from Sigma. The drug was dissolved in DMSO to prepare 1 mM, 10 mM, and 50 mM stock solutions. Three different experimental concentrations of 1 μM, 10 μM, and 50 μM were used to treat the zebrafish embryos. Approximately 30 zebrafish embryos were placed in a 4 cm culture dish, and 3 replicates were set for each concentration. The medium containing E2 was added to the embryos on the second day, and the control group used water containing 0.1% DMSO. The culture medium was replaced with fresh water every 24 h, and on the fourth day, the embryos were placed in 4% PFA for in situ hybridization detection or stored in Trizol at −80 degrees Celsius for RNA extraction and qPCR detection.

### 2.3. In Situ Hybridization

Different developmental stages of zebrafish embryos were fixed with 4% PFA/PBS solution, kept at 4 degrees Celsius overnight, dehydrated with methanol, and stored at −20 degrees Celsius for later use. In situ hybridization was mainly conducted according to the method described by Thisse, C. and Thisse, B [23]. Dual-color in situ hybridization was mainly carried out according to the descriptions of Hauptmann, G. and Jowett, T [24,25]. For in situ hybridization, partial sequences of different genes were amplified and cloned into PCS2+ plasmids. These constructed plasmids were linearized. A T7 Transcription Kit (Roche., Basel, Switzeland) was used for probe synthesis. The primers used for in situ hybridization are listed in the Table 1.

### 2.4. Preparation of Transgenic and esr2b Knockout Zebrafish

The JASPAR database was used for transcription factor binding analysis (https://jaspar.elixir.no/, 16 September 2020). The sequence information of the *GnIH* gene was obtained from the Vega database. *GnIH* promoter fragments with lengths of 2.4 kb (−2357 to +50) were obtained. The amplified *GnIH* promoter fragments were connected to the Psk-CMV + mCherry plasmid using XhoI and ClaI enzymes. This vector has a transposase binding site and fluorescence label. The *GnIH* promoter sequence was then sequenced for validation. The plasmids containing the *GnIH* promoter sequence (100 ng/μL–200 ng/μL) were mixed with phenol red and injected into zebrafish embryos at the one-cell stage using a PLI-90 microinjector (Harvard, Cambridge, MA, USA). Approximately 400 embryos were injected, and the fluorescence location was observed and recorded after the embryos were raised to the three-day post-fertilization stage. The equipment used for photography and fluorescence observation was a Nikon digital sight DS-5Mc camera (Nikon Corporation, Tokyo, Japan) attached to an Olympus fluorescence acro-microscope MVX10 (Olympus Corp., Tokyo, Japan).

Because the 2.4 kb *GnIH* promoter had the best fluorescence intensity and accuracy after injection, we chose it to prepare the transgenic zebrafish lines. The method for preparing transgenic zebrafish lines followed the procedure described by Song et al. [26]. The plasmid containing the 2.4 kb *GnIH* promoter (50 ng/uL), the Tol2 transposase (100 ng/uL), and phenol red were mixed together and injected into zebrafish one-cell stage embryos. After the embryos developed for two days, luminescent embryos were screened and raised to maturity. F1 generation transgenic zebrafish were obtained by crossing them with wild-type zebrafish, and luminescent individuals were selected and raised to maturity for crossing to produce F2 generation transgenic zebrafish. F2 generation transgenic zebrafish were self-crossed to obtain F3 generation homozygous individuals.

The TALENs method was used for zebrafish *esr2b* knockout. For details refer to Peng [19]. mRNA was mixed at a concentration of 100 ng/uL, and phenol red was added before microinjection. After the zebrafish developed to 48 hpf, 5–10 zebrafish embryos were collected, and DNA was extracted for identification of the mutation. The P0 generation embryos were raised to maturity (about 3 months) and crossed with wild-type zebrafish to obtain F1 generation knockout zebrafish. The tails of the F1 generation zebrafish were cut off, and DNA was extracted to identify the type of mutation. F1 generation female and male zebrafish with the same type of mutation were selected and crossed to obtain F2 generation knockout zebrafish. The tails of F2 generation knockout zebrafish were cut off, and DNA was extracted to identify the homozygous individuals with *esr2b* gene knockout.

### 2.5. Real-Time PCR

The Ultra-Pure Total RNA Extracting Kit (Simgen, Hangzhou, China) was used for RNA extraction. The zebrafish RNA was checked for purity and integrity using gel electrophoresis and a spectrophotometer (Thermo, Waltham, MA, USA), respectively. DNaseI was used to eliminate contaminating genomic DNA prior to reverse transcription. The cDNA Synthesis Master Mix Kit (Simgen, Hangzhou, China) was chosen for cDNA synthesis. Briefly, 1 μg of RNA was reverse-transcribed, and the resulting cDNA was diluted five fold for qPCR analysis. Next, 2 × One Step SYBR Green RT-qPCR Mix (Simgen, Hangzhou, China) was used for qPCR, which was performed on a Bio-Rad CFX96 (Bio-Rad, Hercules, CA, USA). The 20 μL PCR reaction contained 10 μL of SYBR mix, 6.4 μL of deionized water, 0.8 μL of forward primer, 0.8 μL of reverse primer (primers listed in Table 1), and 2 μL of cDNA. The qPCR program consisted of 40 cycles of 95 °C for 15 s, 60 °C for 15 s, and 72 °C for 45 s. β-actin was used as an internal reference, and the 2^−△△ct^ method was used to calculate gene expression levels.

### 2.6. Statistics

All estrogen exposure experiments were repeated 3 times. The results are presented as mean ± standard error of the mean (SEM) values. Statistical analyses were performed using SPSS 10.0 and *p* < 0.05 was considered statistically significant. Target gene expression levels were normalized to the expression of β-actin mRNA. All data were analyzed using a Student’s *t*-test or one-way analysis of variance (ANOVA), with multiple comparisons of means performed using Tukey’s test method.

## 3. Results

### 3.1. Expression of GnIH During Embryonic Development

**Figure 1:**
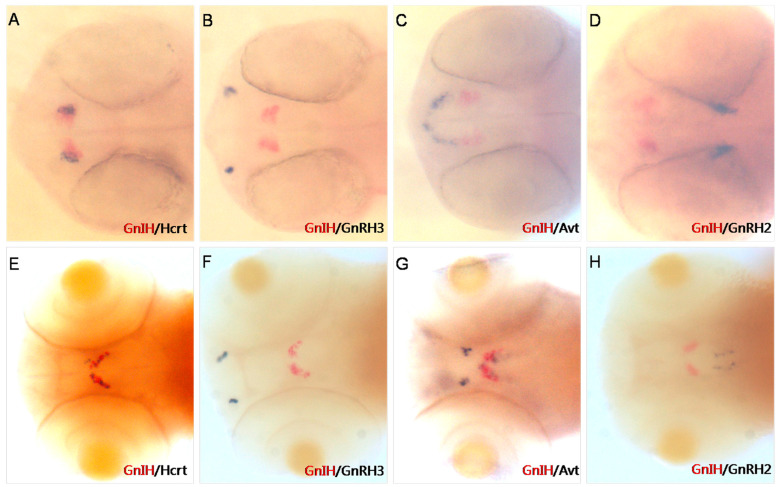
Two-color in situ hybridization detection of *GnIH*, Hcrt, GnRH3, Avt, and GnRH2 (dorsal view) at 36 hpf (**A**–**D**) and 72 hpf (**E**–**H**). Other peptides mark different areas in the brain. At 36 hpf and 72 hpf, Hcrt is expressed in the lateral hypothalamus (**A**,**E**); GnRH3 mRNA is localized in the vicinity of the developing olfactory organ at 36 hpf (**B**). At 72 hpf, it migrates to the transitional area between the olfactory organ and olfactory bulb (**F**); Avt mRNA is localized in the pre-optic area (front of *GnIH* signals) and ventral hypothalamus (close to *GnIH* mRNA) (**C**,**G**); GnRH2 mRNA is expressed in the lateral midbrain at 36 hpf and 72 hpf (**D**,**H**).

To accurately determine the expression location of *GnIH* in early zebrafish embryonic development, we performed double-color in situ hybridization of *GnIH* and other neuropeptides in the brain (Figure 1). The results of double-color in situ hybridization showed that at 36 hpf, the expression of *GnIH* and Hcrt was located in the same position in the zebrafish brain (Figure 1A), while at 72 hpf, the expression of *GnIH* and Hcrt was staggered in the lateral part of the hypothalamus of zebrafish (Figure 1E). Additionally, this study found that at 36 hpf, the expression of *GnIH* was located anterior to that of Avt (Figure 1C), and by 72 hpf, the expression of Avt in the hypothalamus was adjacent to the expression of *GnIH* (Figure 1G). However, in early zebrafish embryonic development, the expression location of *GnIH* was not in the same region as reproductive-associated factors GnRH2/3 (Figure 1B,D,F,H).

### 3.2. Establishment of a Zebrafish Line Expressing a Red Fluorescent Signal Under the GnIH Promoter

**Figure 2:**
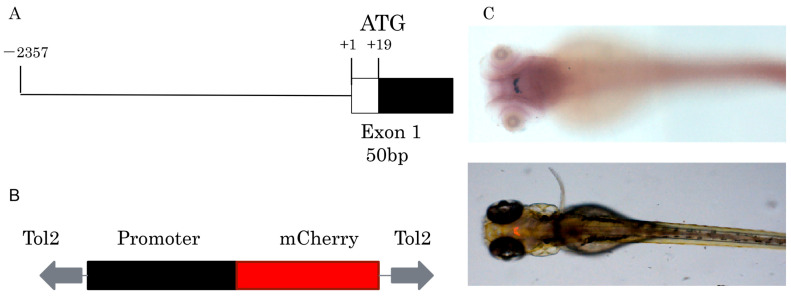
Construction of Tg (*GnIH*:mCherry) transgenic line. (**A**) The fragment used to drive mCherry fluorescence contains 2357 bp of 5′-flanking region and 50 bp of exon1. (**B**) The main elements of the plasmid used for generation of the transgenic zebrafish line, the plasmid contains tol2 elements and mCherry, the promoter elements were replaced by *GnIH* promoter fragments. (**C**) In situ hybridization of *GnIH* mRNA and mCherry fluorescence signal in transgenic zebrafish at 72 hpf (dorsal view).

**Figure 3:**
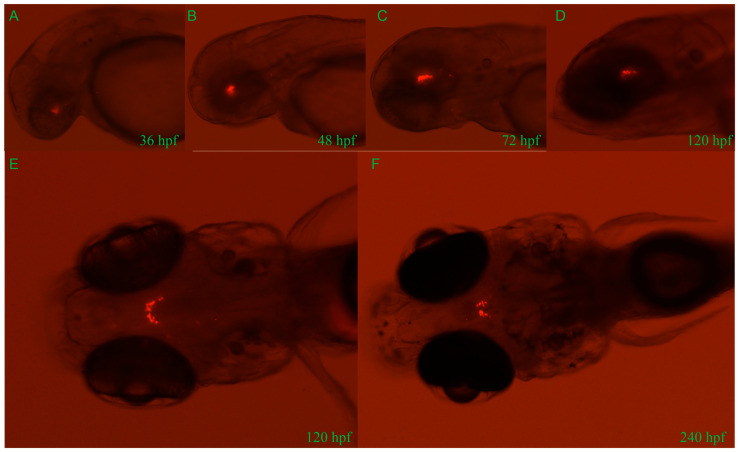
Developmental profile of mCherry fluorescence in the Tg (*GnIH*:mCherry) zebrafish line from 36 hpf to 240 hpf (10 dpf). Lateral views of mCherry fluorescence from 36 hpf to 120 hpf (**A**–**D**). From 36 hpf to 120 hpf, the fluorescence migrates to the lateral hypothalamus. Dorsal views of mCherry fluorescence at 120 hpf (5 d) and 240 hpf (10 d) (**E**,**F**). Consistent with results of in situ hybridization, all fluorescence is restricted in the area of brain.

Approximately 2.4 kb length upstream of the *GnIH* gene was cloned for construction of transgenic zebrafish line Tg (*GnIH*:mCherry), which contains the non-coding sequence upstream of the *GnIH* gene 2357 bp and 50 bp of the first exon’s sequence (Figure 2). The results of in situ hybridization showed that the expression pattern of endogenous *GnIH* mRNA was consistent with the mCherry fluorescence signal (Figure 2C). Subsequently, the fluorescence signals during zebrafish embryonic development were recorded from 36 hpf to 240 hpf (Figure 3). At 36 hpf, specific red fluorescence signals were firstly observed in the brain of transgenic zebrafish embryos. From 48 hpf to 120 hpf, mCherry fluorescence of *GnIH* mRNA continued to be clear and specific in zebrafish brain. The fluorescence signal was still clear and stable at 240 hpf. The fluorescence signals were never detected outside the unique area, indicating that the *GnIH* transcripts were restricted to the lateral hypothalamus, just like Hcrt. From 36 hpf to 72 hpf, the signals were notably enhanced, accompanied by signal migration.

Then, JAPAR, an online database, was used for screening potential transcription factors. The result of transcription binding factor analysis showed that there exists 12 potential estrogen receptor binding sites (Supplementary File S1).

### 3.3. The Effect of Estrogen on GnIH Expression

**Figure 4:**
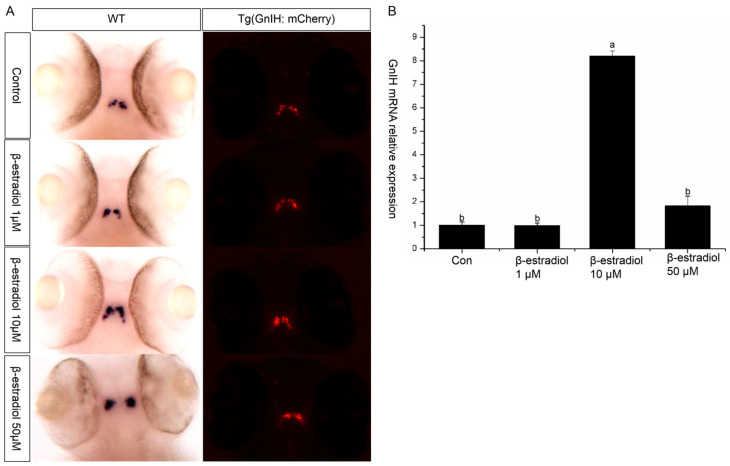
β-estradiol treatment experiments. In the treatment experiments, each treatment group contains 30 embryos (3 parallel). All samples were treated from 48 hpf to 96 hpf. At 96 hpf, in situ hybridization, qPCR, and fluorescence signals were detected. (**A**) In situ hybridization to detect *GnIH* mRNA changes of wild-type zebrafish embryos treated with different doses of β-estradiol (1 μM, 10 μM, 50 μM). This treatment experiment was also repeated using transgenic zebrafish embryos. (**B**) In the wild-type zebrafish embryo treatment experiments, qPCR was also preformed to confirm the results. The results are presented as mean ± SEM values. Values accompanied by different letters are significantly different (ANOVA followed by Tukey’s test, *p* < 0.05).

To explore the regulatory role of estrogen on *GnIH*, wild-type and transgenic zebrafish embryos were exposed to estradiol for 48 h from 48 hpf, with exposure maintained until 96 hpf. Then, in situ hybridization and qPCR were performed to detect the expression changes of *GnIH* in the wild-type zebrafish, and images were captured to record the fluorescent signals in the transgenic zebrafish. The results of in situ hybridization showed that 1 μM and 50 μM had no significant effect on the *GnIH* mRNA content, while 10 μM significantly increased the level of *GnIH* mRNA (Figure 4A). The results of treating transgenic zebrafish embryos with estradiol also showed that the expression level of red fluorescence in the transgenic zebrafish embryos treated with 10 μM estradiol increased significantly (Figure 4A). The qPCR results showed that 1 μM estradiol had no effect on the expression of *GnIH* mRNA, 10 μM estradiol could significantly increase the expression of *GnIH* mRNA, and 50 μM estradiol could promote the expression of *GnIH*, but there was no significant difference compared with the 1 μM treatment group (Figure 4B).

### 3.4. The Effect of Estrogen Receptor 2b Knockout on GnIH Expression

**Figure 5:**
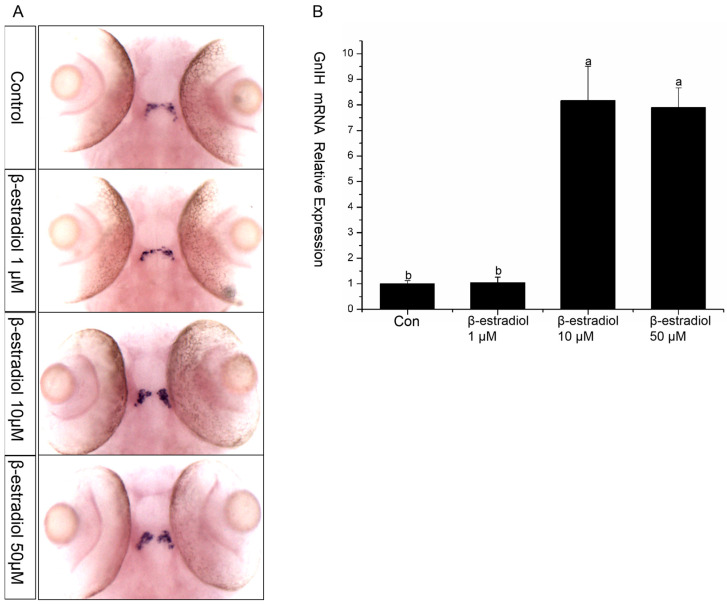
*GnIH* mRNA changes in *esr2b* knockout zebrafish embryos in the β-estradiol treatment experiments. (**A**) In situ hybridization to detect *GnIH* mRNA changes of *esr2b* knockout zebrafish embryos treated with different doses of β-estradiol (1 μM, 10 μM, and 50 μM). (**B**) qPCR results of the treatment experiments. The results are presented as mean ± SEM values. Values accompanied by different letters are significantly different (ANOVA followed by Tukey’s test, *p* < 0.05).

**Figure 6:**
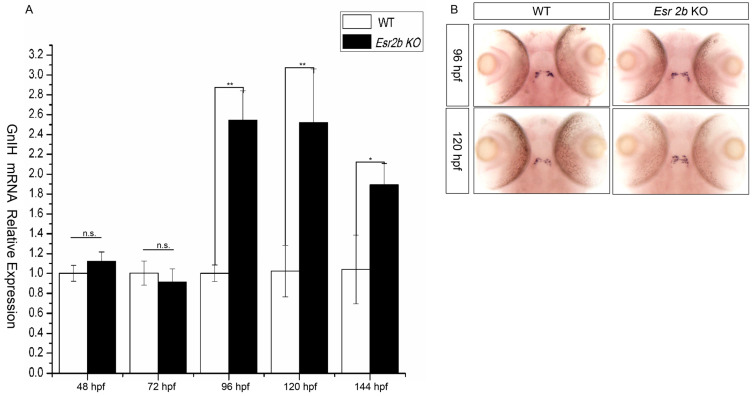
The difference in *GnIH* mRNA expression between wild-type and *esr2b* knockout zebrafish at different developmental stages. (**A**) qPCR was also performed to detect the difference in *GnIH* mRNA expression level from 48 hpf to 144 hpf. (* represents a significant difference, *p* < 0.05; ** represents a significant difference, *p* < 0.01). (**B**) In situ hybridization to detect between wild-type and *esr2b* knockout zebrafish embryos at 96 hpf and 120 hpf. At different developing points, 30 embryos of wild-type and *esr2b* knockout zebrafish were sampled for in situ hybridization and qPCR detection.

To further investigate the regulatory effect of estrogen on *GnIH*, *esr2b* knockout zebrafish were obtained using the TALENs method. Subsequently, we treated 48 hpf *esr2b* knockout zebrafish embryos with 1μM, 10 μM, and 50 μM estradiol for 48 h. In situ hybridization and qPCR were used to detect the expression of *GnIH* (Figure 5). The results showed that, similar to wild-type zebrafish embryos, 1 μM estradiol had no significant effect on *GnIH* expression, while 10 μM and 50 μM estradiol significantly promoted *GnIH* expression (Figure 5A,B). In addition, the expression levels of *GnIH* in wild-type and *esr2b* knockout zebrafish at different developmental stages were detected using qPCR and in situ hybridization. The qPCR results showed that there was no significant difference in *GnIH* expression between wild-type and *esr2b* knockout zebrafish embryos at 48 hpf and 72 hpf. However, at 96 hpf, 120 hpf, and 144 hpf, the expression of *GnIH* in *esr2b* knockout zebrafish embryos was significantly higher than that in wild-type embryos (Figure 6A). The in situ hybridization results also showed that at 96 hpf and 120 hpf, the expression of *GnIH* in *esr2b* knockout zebrafish was significantly higher than that in wild-type zebrafish (Figure 6B).

## 4. Discussion

As a neuropeptide that negatively regulates reproduction, the early development of *GnIH* is still poorly understood. Immunohistochemical results in zebrafish showed that the *GnIH* protein signal is present in the hypothalamus at 37 hpf [27]. Zhang et al. used RT-PCR to monitor the expression of *GnIH* during zebrafish embryonic development and found that zebrafish *GnIH* began to be expressed at the prim-5 stage [7]. In our previous work, in situ hybridization results showed that the *GnIH* signal appeared in zebrafish at 24 hpf. In the present work, *GnIH* had a specific distribution in the zebrafish brain during early development, showing a symmetrical distribution (Figure 1). Dual-color in situ hybridization results showed that *GnIH* was expressed in a position that overlapped with *Hcrt* expression in the early stage, located in the ventral midbrain of the hypothalamus, also known as the lateral hypothalamus [28]. In vertebrates, the brain has functional divisions. The expression position overlap indicated that *GnIH* and *Hcrt* may perform similar functions. The *Hcrt* gene encodes neuropeptide that is involved in the regulation of sleep and energy balance in mammals [28]. However, *GnIH* signaling and *GnIH*-expressing neurons are both necessary and sufficient to promote sleep, mature peptides derived from the *GnIH* preproprotein promote sleep in a synergistic manner, and stimulation of *GnIH*-expressing neurons induces neuronal activity levels consistent with normal sleep [29]. In addition, the zebrafish prepared in this study with the transcribed *GnIH* promoter accurately marked the activity of endogenous *GnIH*. By tracking the fluorescence labeling of transgenic zebrafish embryos, we found that *GnIH* fluorescence signals were concentrated in the lateral hypothalamus and were highly specific. The signals were first observed at 36 hpf (Figure 3). Currently, zebrafish mainly contain three nucleus estrogen receptors: *esr1*, *esr2a*, and *esr2b*. Research on the three estrogen receptors in zebrafish embryos showed that in the early stage of zebrafish embryonic development, the content of *esr2a* mRNA was high in newly fertilized oocytes of zebrafish, the content of *esr2a* mRNA gradually decreased and disappeared between 6 hpf and 12 hpf of zebrafish embryonic development, and when the zebrafish developed to 24 hpf, the homozygous gene was activated, and the content of *esr2a* mRNA began to rise again [30,31]. Unlike *esr2a*, the expression level of *esr2b* was very low in newly fertilized oocytes of zebrafish, and the expression levels of the two receptors increased significantly as zebrafish embryos developed [30,32]. Overall, during the 12 hpf–48 hpf developmental period of zebrafish embryos, the mRNA expression levels of the three estrogen receptors were relatively low, and the content of estrogen receptor mRNA increased significantly from around 48 hpf [33]. In situ hybridization results of zebrafish embryos showed that the content of *esr2a* and *esr2b* mRNA increased significantly from 48 hpf to 60 hpf, and most of the signal was located in the hypothalamus area [34]. In contrast to *esr2a* and *esr2b*, *esr1* mRNA had a relatively low content in the early stage of zebrafish embryo development, mainly located in the liver [34]. Estrogen receptors were involved in regulating *cyp19a1b* during zebrafish embryo development. A low dose of estradiol (10^−8^ μM) was able to trigger *cyp19a1b* expression in the preoptic area and mediobasal hypothalamus at 48 hpf and 108 hpf [35]. The cyp19a1b-gene is so sensitive to estrogens that it has been proposed as a biomarker for estrogenic exposure. It was reported that the *cyp19a1b* promoter containing an estrogen-responsive element (ERE) located upstream of the transcription start site is absolutely mandatory for upregulating aromatase expression by estrogens [35]. This evidence indicated that estrogen receptors are involved in neurogenesis during zebrafish development [36]. Meanwhile, estrogen receptors *esr2a* and *esr2b* are widely present in the hypothalamus of zebrafish embryos in the early stage of development, consistent with the expression location of *GnIH*, and early expression of *GnIH* is likely to be regulated by estrogen.

Currently, there is very limited research on the regulation of *GnIH* by estrogen. In mice, Molnár et al. implanted capsules containing estradiol under the skin of female mice that already had their ovaries removed, and after four days of treatment, they found a significant decrease in the amount of *GnIH* mRNA [22]. In addition, researchers found that estrogen receptor alpha (ERα) is expressed in *GnIH* neurons [9]. In hamsters, it was also found that about 40% of *GnIH* neurons express ERα, and treatment with estradiol in hamsters can enhance *GnIH* neuron secretion activity [9]. These pieces of evidences suggest that estrogen regulates the expression of *GnIH* through ERα. In this study, we conducted a transcription factor binding analysis of *GnIH* promoter sequence fragments and found potential estrogen receptor binding sites on the sequence. Subsequently, we treated wild-type zebrafish embryos and transgenic zebrafish embryos (Tg *GnIH*: mCherry) with estradiol and found that 1 μM estradiol had no effect on *GnIH* expression, while 10 μM estradiol significantly increased *GnIH* expression, and 50 μM estradiol increased *GnIH* expression, but not significantly. These results indicate that estrogen is able to promote the expression of *GnIH* during the early stage of zebrafish development. *GnRH* is a classic neuropeptide involved in regulating vertebrate reproduction. In both males and females, gonadal steroid hormones exert negative feedback regulation on axis activity at the levels of both the pituitary and the hypothalamus [13]. The feedback regulation is mediated by estrogen receptors(ERs). Many studies have shown that estrogen has a bimodal effect on the hypothalamus with both inhibitory and stimulatory influences on GnRH secretion [37,38,39,40,41]. In an in vivo study, there was observed a decrease in GnRH expression and secretion by estradiol in both the GN11 and GT1-7 *GnRH*-expressing cell lines, and these effects were determined to be primarily mediated by ERα in GT1-7 cells and by both ERα and ERβ in GN11 cells [40]. In our work, 10 μM was able to induce *GnIH* transcription. But a high dose of estrogen (50 and 100 μM) lost this function. We hypothesize that the failure of elevated estrogen concentrations to upregulate *GnIH* expression is likely attributable to classical negative feedback regulation. To further study the regulation of *GnIH* by estrogen, *esr2b* knockout zebrafish were constructed. Similarly, *esr2b* knockout zebrafish embryos were treated with estradiol and we found that a 1 μM concentration of estradiol still had no effect on *GnIH* expression, while 10 μM and 50 μM estradiol both significantly promoted *GnIH* expression. Since zebrafish contain three types of estrogen receptors, *esr1*, *esr2a*, and *esr2b*, we hypothesized that after *esr2b* knockout, estrogen may still promote *GnIH* expression through esr1 or esr2a. Interestingly, high concentrations of estradiol (50 μM) did not significantly promote *GnIH* expression in wild-type zebrafish but significantly increased *GnIH* expression in *esr2b* knockout zebrafish. Afterward, we detected the expression of *GnIH* in the development process of *esr2b* knockout zebrafish (48 hpf–144 hpf) and found that there was no significant difference in the expression of *GnIH* in zebrafish embryos at 48 hpf and 72 hpf between *esr2b* knock-out and wild-type zebrafish. However, from 96 hpf, the expression of *GnIH* in *esr2b* knockout zebrafish was significantly higher than that in wild-type zebrafish. Therefore, we believe that *esr2b* may be responsible for the negative regulation of *GnIH* expression, and the establishment of this relationship begins on the fourth day of embryonic development. Our work mainly explored the regulatory role of the estrogen signaling pathway in the early development of *GnIH* in zebrafish by preparing transgenic zebrafish and *esr2b* knockout zebrafish.

This study found that in the early development of zebrafish, *GnIH* expression can be induced by estrogen. At the same time, it is the first to discover the negative regulatory role of *esr2b* on *GnIH* from the fourth day of zebrafish development. Considering the complexity of estrogen receptors, more esr knockout models may be needed in the future to explore the regulatory role of the estrogen signaling pathway on the early expression of *GnIH*.

## 5. Conclusions

In this study, Tg (*GnIH*: mCherry) and *esr2b* knockout zebrafish were used to explore the regulation between estrogen receptors and *GnIH*. Our work showed that the *GnIH* neuropeptide expression location overlaps with that of Hcrt at 36 hpf and 72 hpf. Estrogen treatment experiments showed that an appropriate dose of E2 is able to induce *GnIH* mRNA levels. High-dose E2 (50 μM) is not able to significantly induce *GnIH* mRNA levels. *Esr2b* knockout led to increased *GnIH* mRNA levels in zebrafish embryos and it started on the fourth day. In a word, estrogen receptors are able to regulate *GnIH* expression in the early zebrafish development stage. However the *esr2b* is involved in negative regulation.

## Figures and Tables

**Table 1:** Primers used for probes and qPCR (**protecting group is in bold type**).

Primers Used for Probes and qPCR		Accession No.	Length
F: **CGGGATCCC**GATGTCCTACTTCGCTCTTCT	*GnIH*	NM_001082949.1	598 bp
R: **GCTCTAGAGC**TTAGTCTAAAGCTGTGTAGTCT			
F: GACCGAGCTCCCAAGTCTAC(qPCR)	*GnIH*	NM_001082949.1	124 bp
R: AAATGTTCCTCCTGCCAAAC(qPCR)			
F: ACCAACCATGACACCCTGATGT(qPCR)	*β-actin*	AF025305.1	135 bp
R: CAACGGAAACGCTCATTGC(qPCR)			
F: **CGGGATCCC**TCCAGGTGCTCGTCTTCAT	*hcrt*	DQ831346.1	381 bp
R: **GCTCTAGA**TAGCGACAAGTGTCATCGTTTT			
F: **CGCGGATCC**GTCCAGTTGTTGCTGTTAGTTTG	*gnrh3*	NM_182887.3	344 bp
R: **GCTCTAGATT**GGAGGATATTTCATTAGGAGT			
F: **CGCGGATCC**GGCTCTGTGATTTTACTCAACCG	*gnrh2*	AF511531.1	344 bp
R: **GCTCTAGAG**GGCATCCAGCAGTATTGTCTTG			
F: **CGGGATCC**CAGAACTGCCCACGAGGAGG	*avt*	AY168623.1	374 bp
R: **GCTCTAGA**CTGCGGGTTGCCAGATTGAG			

Note: protecting bases is in bold type.

## Data Availability

All data generated or analyzed during this study are included in this published article and its supplementary information files.

## Supplementary Materials

The following supporting information can be downloaded at: https://www.mdpi.com/article/10.3390/ani16030444/s1, File S1. The analysis of GnIH promoter sequence online(JASPAR database).
