# Supplementary material for: Estrogen Receptor 2b Is Involved in Regulating Gonadotropin-Inhibitory Hormone Expression During Early Development in Zebrafish
Source: Animals (Basel). 2026 Feb 1;16(3):444. doi: 10.3390/ani16030444 (PMC12896573; doi:10.3390/ani16030444)
Supplement: Supplementary file 1 [file animals-16-00444-s001.zip › animals-4100525-supplementary.pdf]

Supplement 1 The analysis of GnIH promoter sequence online(JASPAR database).

tgctgactcgaatggaaatacagattggaatg**acatgaaggtga**gtaattaataacataattgacatttttggtgaataacct

ER

ttagcaaatgaagtataaaatgtcggtcttctccagacagttctgatgacaaaattaaataccttcccctcactaccattgat

catatgcatggcagttatggcaattagcaaatatttttgatagaataaaataccacagtcattctgtggtctgcaattcctcatcc

aggcatgttaacgtc**ttttcaagctca**tgtgtcatacgtcttttagagaaaacatcagaccattctttctcactccatcggtggtat

ER

tg**gggtctcagtgggct**gcacaatgaattgatttgcactattatgtgttgaatgcatctccagctaatttg**gagcaagtcc**ac

ER ER

cttttgttcggtataaacacatggaatagaagatgtgcaaatttccaagggattactggttagccttttccggcttatgctcaattat

aaaaccatgatgaaaaaacaaggtaattgaatacaataatttatcattgatgctttacaagtttaataccagtagctatattaag

aaataatgccaaatggtacaacagaaaaacctgtctctttacattcattgggacttttaaaggtaatctaacctatccagtttttat

ggattgttttacattttgcaaatgttaataagaataagacaataaaaacattttgatatcaatctgcacgtactgtgatcacagtta

tgcccatttttgaaggctcctgtaaattcatactgtgaaaaacagggtgaaaaaaaagctttctttgatctaattcaatta

ER

atcaattgacggtaatggttcacatcaaaagtttattacct**gagca**atgataaatatataatttgccttaatctatattttctatgtc

ER

atgaaaaatcttttaaataatctggtacaaagcatatttgtcttgttgaagaaagtcaattgattatttaaattttcttttgatctaac

aaaaagatacagcttcaaattggtggtccactatgatatcaaattttcaattttagttcatgtgtaatgtaaatgtgtgaacataaa

caacaactctgaatgtaatat**gctcaaagttca**atgcaaagggagacattggcttttacagagttagcttagcaaagcctc

ER

agcaaacaaaatttagggactacaaacaaataaatctgggctagtgagatcacaaacacttcggattacatgcattcaca

agcgcatacaccccgtgcagcaactataatgttatagtactaaaagctaaaatgccgtctaaacgccaccatttccacaga

gcttgttctgtttctggatttgggcttccaaagtacatgacacaaagagagaagtgtttgcaatttaattttaattatggtccagaa

atgaaaaaaaaaaaaaaaaaaaagatatagctagcatttgacaaaagagagcttccagaatctctcctagttcagcgct

agactcagctaaaactcctccaaccatattaaaagaagctgtggattgtgagccacaacctgcaagcatttttatttgctaaaa

t**tgacctattaca**tgcacagtttctagtgttaacggtatgttgtagcaaggatgtattatttagcctgacatgtttactgttccaa

ER

attttaaatgtttcctaaaataaaatatattgttttcaaaggggaacaagtttttgtttatttagacatttaaaaagaatatatttca**g**

agcagtatcacaatactatgatattttttt**tatccaaggtta**tcatatcgtcagaatcctggcccatgtcttttgctcactaatgtaa

ER ER

catatcaatttttcaacactacctaccacgataacctcccacttcacaggcagggcttaaggctagtcccagacttaattaact

gaaaataactagtgatatatctattgagttgccttgcagtaatatgttttctgtataaagttgtttttattttattgtattatccaattaaat

acatttaactgaggcctaatcctggcttaaactgttaaagtattaaaaagaggggtttaagtattaaatcatagtgacttgtcatt

ttctggtgcattaagaagcctaagacgccactggttgctatgaaacac**tcaagaaggtca**ttagcgaat**gaatgaaggtt**a

ER ER

tgctgctggtggagttaatatattaaatatgcaaagctgtgagtgcattataaggaatgtctttgtgatcatgttgatttgcacgtg

cccaaatgtataaaaagcttgtgatgttcctggccttcagtgaccgattgtaagatgtcctacttcgctcttctttctttagccc
